# Supplementary figures and images for: In vivo synthesis of bacterial amyloid curli contributes to joint inflammation during S. Typhimurium infection
Source: PLoS Pathog. 2020 Jul 9;16(7):e1008591. doi: 10.1371/journal.ppat.1008591 (PMC7347093; doi:10.1371/journal.ppat.1008591)

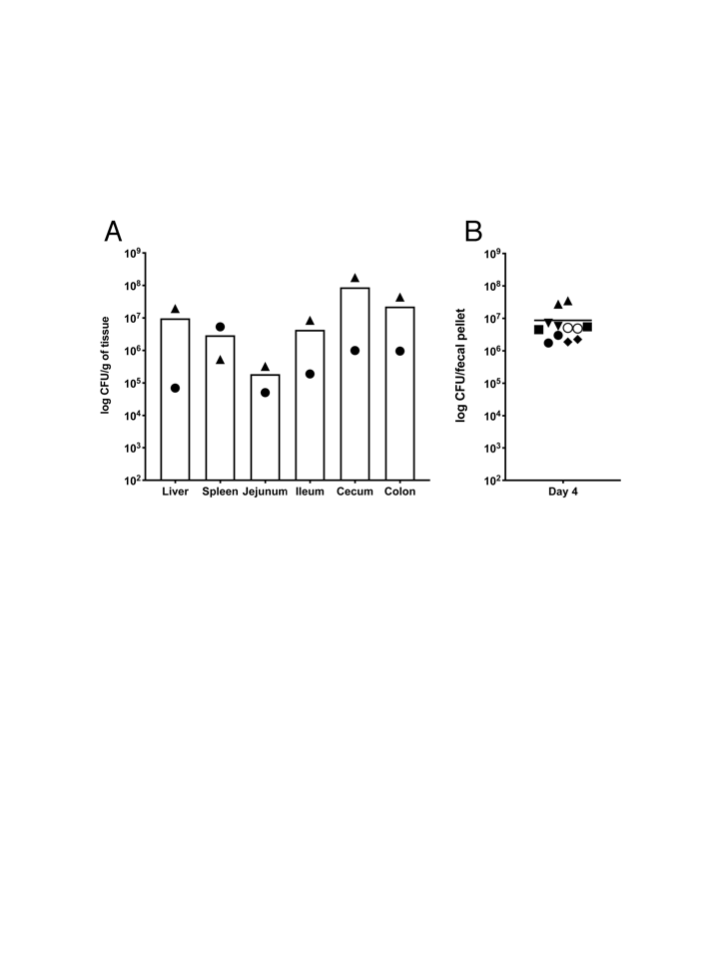

Supplement: S1 Fig — Organs were collected and bacteria enumerated from two mice from a group of six that were infected with S. Typhimurium 14028. (A) Arrowheads and circles represent the values from each organ for each mouse analyzed, and bars represent the mean values. (B) Two fecal pellets were collected from all six infected mice on Day 4 post-infection and the levels of S. Tm were determined. Each symbol type represents a different mouse. (TIFF) [file ppat.1008591.s001.tiff]

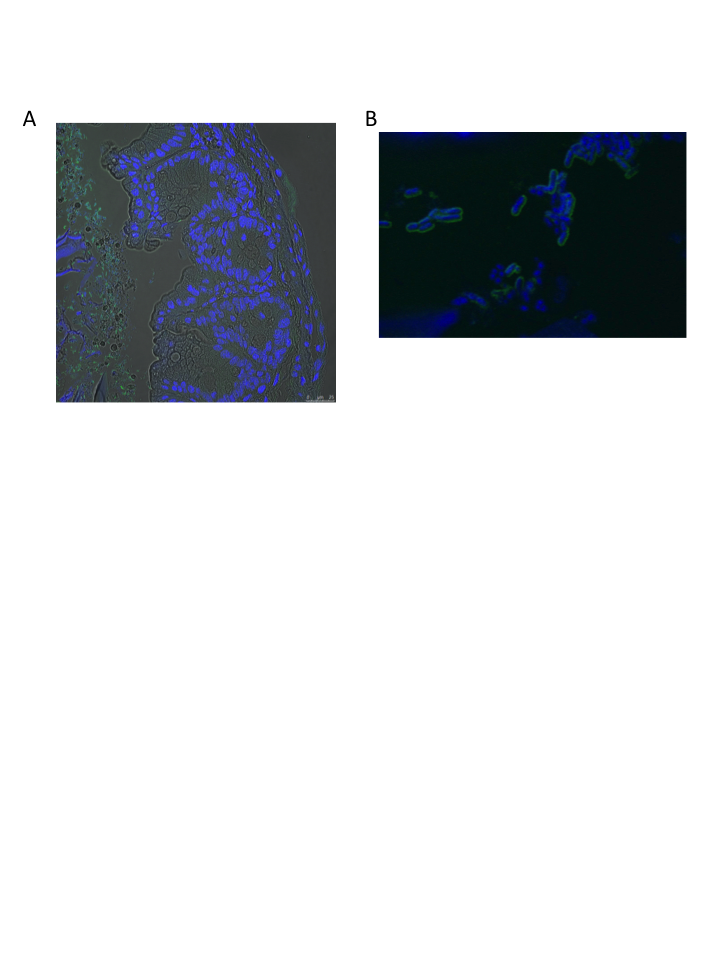

Supplement: S2 Fig — Streptomycin pre-treated C57BL/6mice were orally infected with 108 CFU of S. Typhimurium. Organs were collected 96 hours post-infection, fixed in 10% formalin and paraffin embedded. 5 μm sections were cut and stained with DAPI for nuclear staining or with Salmonella anti-04 LPS antibodies. FITC-conjugated anti rabbit IgG was used as secondary antibody. (A) Cecal tissue was visualized at 60X. (B) Lumenal bacteria from the cecal tissue was visualized at a higher magnification (100X oil). (TIFF) [file ppat.1008591.s002.tiff]

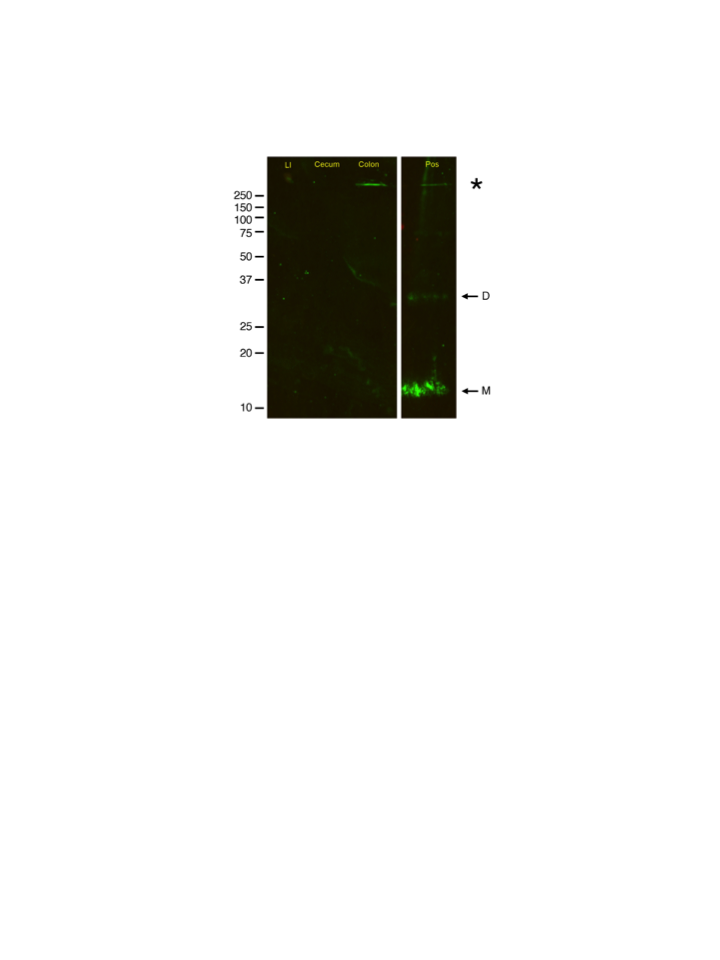

Supplement: S3 Fig — Liver (LI), cecum and colon tissues were harvested from S. Typhimurium-infected C57BL/6 mice and boiled in SDS-PAGE sample buffer. The insoluble debris remaining was washed with distilled water and treated with 90% formic acid prior to SDS-PAGE and immunoblotting. Purified curli were included as a control (Pos), with arrows denoting the CsgA monomer (M) and dimer (D) species. The star represents the high molecular weight species associated with the top of the SDS-PAGE well, present in the colon sample and the positive control. Molecular mass markers (in kilodaltons) are indicated on the left. Curli bands were detected with rabbit anti-curli immune serum as primary antibody and IRDye 680RD Goat-anti-rabbit-IgG (LI-COR) as secondary antibody; immunoblots were visualized on an Odyssey CLx scanner (LI-COR). (TIFF) [file ppat.1008591.s003.tiff]

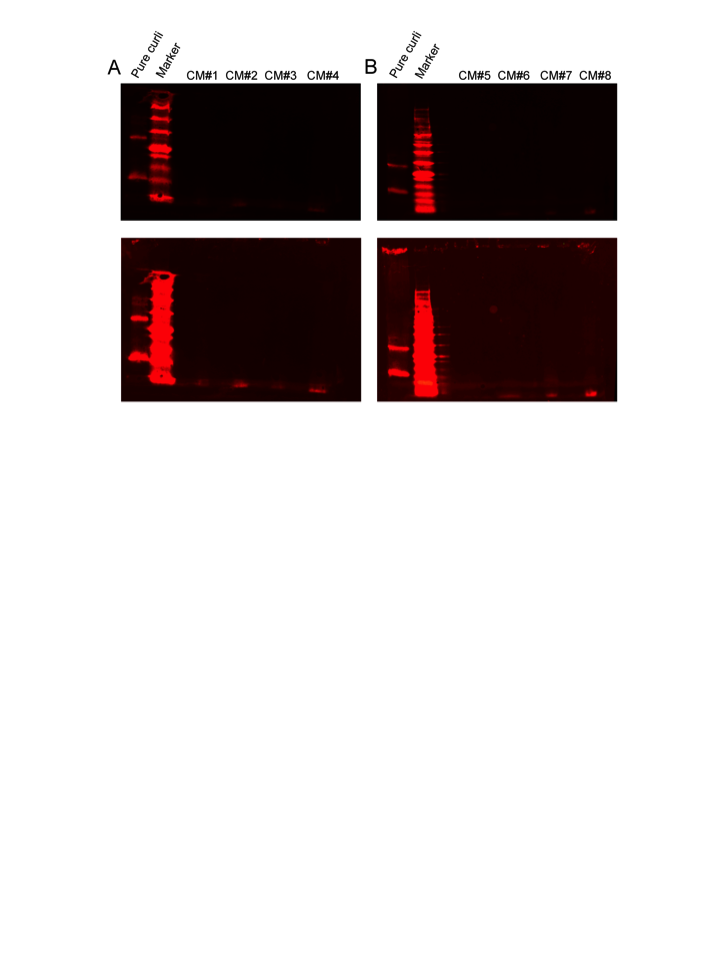

Supplement: S4 Fig — Colon samples from 8 control mice (CM#1–8; A and B) that do not contain Salmonella, were processed for curli production. Samples were frozen, ground, boiled in SDS-PAGE sample buffer and the insoluble debris remaining was treated 3x successively with 90% formic acid before running the gel. Formic acid-treated, purified curli samples were included as controls (Pure curli). Rabbit anti-curli immune serum was used as the primary antibody, followed by IRDye 680RD goat anti-rabbit immunoglobulin G (IgG) secondary antibody and visualization using the Odyssey CLx imaging system and Image Studio 4.0 software package (Li-Cor Biosciences). Representative images are shown; on top, the auto-exposed images generated by the Odyssey are shown, whereas, on bottom, the same blots have been over-exposed to show that there are no faint curli bands present. (TIFF) [file ppat.1008591.s004.tiff]

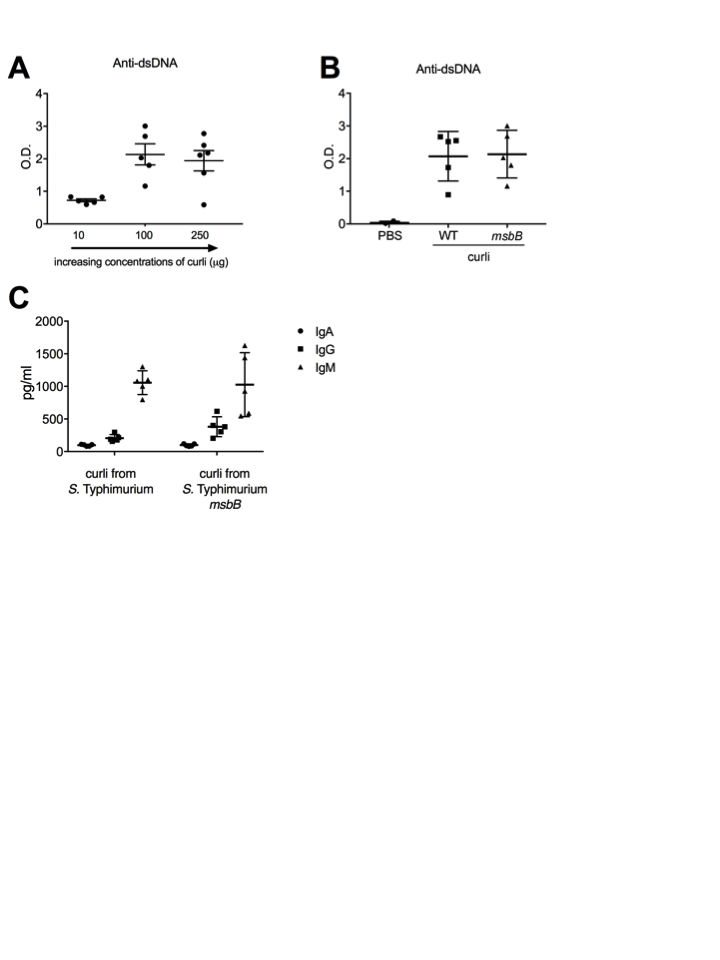

Supplement: S5 Fig — Balb/C mice were immunized intraperitoneally with 10, 100 and 250 μg of purified curli (A) or with 100 μg of curli purified from wildtype S. Typhimurium or an isogenic msbB mutant (B). Injections were continued weekly for 4 weeks. At the end of 4 weeks, the levels of anti-dsDNA autoantibodies in the serum of mice were quantified by ELISA. (C) Levels of IgA, IgG and IgM in the serum of mice from (B) were quantified by ELISA. (TIFF) [file ppat.1008591.s005.tiff]
