# Supplementary material for: In vivo synthesis of bacterial amyloid curli contributes to joint inflammation during S. Typhimurium infection
Source: PLoS Pathog. 2020 Jul 9;16(7):e1008591. doi: 10.1371/journal.ppat.1008591 (PMC7347093; doi:10.1371/journal.ppat.1008591)
Supplement: Attachment — Submitted filename: Miller et al PP point-by point responsefinal.docx [file ppat.1008591.s006.docx]

**MS ID#: PPATHOGENS-D-20-00087**

**MS TITLE: *In vivo* synthesis of bacterial amyloid curli contributes to joint inflammation during *S.* Typhimurium infection**

We thank the Editors of PLOS Pathogens for considering our manuscript for publication. Moreover, we also thank all reviewers for their comments and suggestions. We have addressed the comments and revised the manuscript. We think these changes have improved the manuscript significantly as a result.

Please, find below our point by point answers to all the criticisms of the reviewers.

**Reviewer's Responses to Questions

Part I - Summary**
Please use this section to discuss strengths/weaknesses of study, novelty/significance, general execution and scholarship.


**Reviewer #1:** This study by Miller et al demonstrates that invasive Salmonella can promote arthritis “like” responses in infected mice through their expression of curli. This is explored through the use of mouse models of Salmonella infection, in vivo imaging and blotting for curli expression, as well as assessments of immune responses and tissue inflammation. Overall, the findings are largely convincing, and the implications are significant, considering the widespread interest regarding the potential for gut microbes to promote systemic autoimmune diseases. Even so, there are some additional experiments, revisions to the text and clarifications needed before the manuscript should be accepted for publication.

RESPONSE: We thank the reviewer for appreciating the significance of this work.

**Reviewer #2:** The role and importance of curli in biofilm formation and host colonization is well-documented. This is a well-written manuscript with moderately novel and at the same time thought-provoking findings. The authors claim that Salmonella produces curli during experimental mice infection and that curli is responsible for inducing autoimmunity through anti-ds DNA auto-Abs. The authors reference their own paper in which a curli-expressing Salmonella induces autoimmunity generating anti-ds DNA (ref. 36). Previous studies by Crawford R. (PNAS) showed the Salmonella produces biofilms in the gall bladder in humans, which suggest that curli is produced in the human GI tract. The present work sounds like a confirmation of previous findings.

RESPONSE: We thank the reviewer for this cogent summary of our work. Although the previous studies from Crawford et al suggested that biofilms of Salmonella exist on gallbladder of Typhoid patients, these studies have not reported a role for curli on gallstone biofilms. Two other papers from John Gunn’s lab have indicated that curli is not part of the gallstone biofilms (Crawford et al. 2008, PMID: 18794278; Marshall et al., 2014, PMID: 24551241). Moreover, typhoid strains of Salmonella (not strains causing gastroenteritis) have pseudogene formation or gene degradation in the csg cluster that encodes curli (MacKenzie et al., PMID: 31233504) suggesting that the composition of biofilms associated with gallstones could be different than those associated with the gut. To date no studies have explored the expression of curli in the intestinal tract.

Our previous study where we showed that curli/DNA complexes or biofilm material could lead to an autoimmune response was conducted by introducing purified curli or biofilm material through intraperitoneal injections, by-passing the gut. In this study we show that gut presence of curli/DNA complexes or biofilm is not enough to elicit an autoimmune response. An invasive infection or a leaky barrier is required to elicit such autoimmunity. Furthermore, we take our previous findings a step further showing that presentation of curli systemically following the invasive Salmonella infection leads to joint inflammation. Overall we feel that these findings provide a novel mechanistic explanation for the onset of reactive arthritis following Salmonella infection. Because curli is also produced by many members of the family Enterobacteriaceae (e.g., *Shigella, E. coli*), our results will likely provide insight into the autoimmune complications and reactive arthritis triggered by other enteric pathogens.

**My specific comments are:**

Fig.1 A Salmonella curli mutant and a complemented strain is missing in this experiment.
RESPONSE: In the original figure an isotype control, naïve rabbit sera, serves as a control staining to demonstrate specificity of staining with the curli specific anti-sera. This application of an isotype control is generally recognized as an appropriate control for use in immunohistochemistry. Even with this control, it is possible that the antibodies generated besides those that are specific curli can lead to nonspecific staining. It is for this reason we concluded that additional experimentation was needed to determine whether curli was produced in the gut in vivo. We reasoned that additional expression studies together with the western blots from various samples were required to justify the conclusions. Nevertheless, to address the reviewers point, we utilized our anti-curli polyclonal serum and stained intestinal sections from mice infected with the curli mutant strain. We detected positive staining with the polyclonal serum in the sections from mice as discussed above (see below).

Salmonella (green) and Curli (red) along with DAPI counterstain (blue). Wild type (Left image), curli mutant (right image)

The complemented strain suggested by the reviewer would also be a good idea, however, due to the regulatory complexities controlling curli expression, the complemented strains are never ideal. Previously, we compared a chromosomal complemented strain and a plasmid complemented strain (Shivak et al., 2016, PMID: 27260360), but in each case, curli gene expression did not match normal physiological levels in a wildtype strain. Therefore, we feel that an infection experiment would not give clear results.

Line 119. I suggest to write “ 25% of the bacteria” and not “25% of cells”.

RESPONSE: We changed the wording.

Line 124. Maybe instead of saying “individual” the sentence should read “ a very small number (how many?) of single bacteria”….

RESPONSE: Thank you for the suggestion. We removed “individual” from this sentence.

Line 185 and Fig. 4. The data in Fig. 4 suggest that curli does not promote Salmonella colonization in the gut, liver or spleen. It’s well known that curli is an important component of biofilms of Salmonella and pathogenic E. coli and that biofilm formation is key for colonization and persistence of bacteria in the gut and other host tissues and surfaces. I think the authors need to highlight this finding because it challenges the current knowledge on the role of curli. I did not see this in the discussion section either. Why is curli not important for colonization but important for biofilm formation?

RESPONSE: We agree with the reviewer that this is an important observation regarding the Salmonella biofilms. We have previously reported that curli are not important for colonization, in fact in some situations they can be a disadvantage (Nishimori et al. 2012, PMID: 23027540; White et al. 2008, PMID: 18195033). Since this finding was reported before we didn’t include it in the discussion. However, we have now highlighted these studies as consistent with our findings in the results section Line 185. discussing Fig 4.

It should also be noted that in this study we found no evidence of curli production in bacteria found outside of the GI tract, See figure 1 E and H. This observation fits with our previous published results.

Panels 4E and 5C. What does Arbitrary Units mean? Why not titers of Abs or Elisa ODs? Why was the presence of IgA Abs not tested in gut contents and sera? This is a very important question.
RESPONSE: We thank the reviewer for pointing this out about arbitrary units. This was actually a mistake on our end. The measurements are ODs for the anti-dsDNA autoantibodies. We apologize for this oversight and corrected it in Figures 4E and 5C.

We thank the reviewer for the recommendation of testing IgA in the gut contents and sera. We didn’t initially think of this because our studies were initiated with looking at the systemic pathogenic response to curli (Gallo and Rapsinski et al. 2015 Immunity, PMID: 26084027). We were interested in determining whether curli associated with DNA would elicit an autoimmune response and antidsDNA autoantibodies as reported for amyloid/DNA complexes. Once we determined that curli/DNA complexes elicit anti-dsDNA autoantibodies with mostly subclasses IgG2a and 2b, we concentrated our efforts using IgG autoantibodies as a proxy for immune responses for curli. Since we show now the expression of curli in the intestinal tract, we agree that it is critical to determine the IgA responses. We started looking at these responses upon receipt of the critiques; we established a new protocol and obtained some preliminary results. We determined that 4 days post-infection, there was an increase in the curli specific IgA in mice that were infected with wild type Salmonella compared to the mice infected with the curli mutant (csgBA) or the mice injected i.p. with purified curli for 4 weeks. We would have liked to do the same ELISA from fecal samples from mice infected with the bacteria longer than 3 weeks as antibodies would take time to be developed. We started these experiments but due to the COVID-19 outbreak, they were terminated. We hope to address these comments in a future study as it would require more detailed studies.

Line 247. It is not a natural infection. This is an experimental infection.
RESPONSE: We corrected this in the manuscript and deleted “natural” .

Line 314. What is the basis to hypothesize that individuals that carry the HLA-B27 react stronger to bacterial curli, which may help justify the occurrence of ReA after GI infections? Are there supporting data here?

RESPONSE: Very little is known about the explicit mechanisms that potentiate the development of autoimmunity. However, it is generally accepted that susceptibility to autoimmune diseases is multifactorial, and that the interaction between genetic and environmental factors underlies disease progression. Different genetic factors are associated with susceptibility to autoimmune diseases, and various environmental factors feed into breaking tolerance to self-antigens in the susceptible host. One such factor is HLA-B27, as individuals carrying this allele exhibit an increased risk for developing ReA. Since we determined that curli-expressing S. Typhimurium or E. coli can potentiate pathogenic autoimmune responses and joint inflammation, we hypothesize that these responses may be exaggerated in individuals with HLA-B27. We plan on testing this hypothesis using transgenic HLA-B27 mice. However, we do not have any preliminary results yet that we could include in the manuscript.

Line 351, Fig. 6F. Choice of E. coli MC4100 not the most adequate one I think. MC4100 is a lab/domesticated strain that has been passed for many years in the lab. Why not a pathogenic E. coli?

RESPONSE: In our previous study Gallo and Rapsinski et al. 2015 Immunity we tested another strain of E. coli, Nissle 1917, and showed that the anti-dsDNA responses were dependent on curli expression. E. coli Nissle 1917 is a probiotic strain but it has LPS and cellulose which could both be confounding factors for the immune responses. In the current study, we utilized E. coli MC4100 because this strain doesn’t have LPS and cellulose, therefore making this strain ideal to study immune responses specific to curli. We also did not want to use pathogenic strains of E. coli to avoid the complications that may arise due to the virulence properties. The study that uses E. coli MC4100 only shows that the location of the curli presentation is important because the autoimmune responses were not detected when the bacteria is in the gut.

*E. coli* MC4100 was also recently used in the recent Sampson et al., 2020 (PMID: 32043464) showing that curli can promote protein misfolding in a mouse model for Parkinson’s disease.

Panel 6F. Please indicate inoculation routes under strains.

RESPONSE: We have now included this information.

Immunization of animals. Nowadays, in most institutions the use of Freund’s adjuvant is not accepted. Please clarify why the use of CFA in immunizations. Do the participating institutions allow it?

The reviewer is correct. Our University animal care committee only approved the use of complete Freund’s adjuvant if it was diluted 1 in 10 in incomplete Freund’s adjuvant, reducing the amount of mycobacterial antigens below 0.1 mg/mL. We have made this point clearer in the main text. The reasoning for using CFA was to be consistent with immunization procedures used by Collinson et al. 1991 (PMID: 1677357), who had generated the only curli-specific polyclonal immune serum ever used by members of the White lab.

**Reviewer #3:** The authors describe the in vivo production of curli amyloid fiber during S. Typhimurium infection in the murine gastrointestinal (GI) tract following oral infection. Using mouse models for Salmonella infection, the authors conclude that the presence of curli in the GI tract is associated with increase in autoimmune disorder as quantified by increase in the production of autoantibodies and inflammation in the infected mice. The authors further claim that the de novo curli synthesis in the GI tract of infected mice could represent a link between GI enteric infections and reactive arthritis.

**Part II – Major Issues: Key Experiments Required for Acceptance**

**Reviewer #1:** 1. A major issue is whether curli are expressed – de novo within the mouse intestine or if curli expression depends on the passage of Salmonella out of the gut, and is induced once they are passed into the external environment in the stool. The gene csgD is clearly transcribed within the mouse gut, but much of the luciferase signal is found in the small bowel (as shown in fig 2). During Salmonella infection, the only Salmonella that should be found in large numbers in the small bowel would be those found in stool that is eaten. Similarly, the fraction of Salmonella that express immunoreactive curli in the gut, could be those that has been ingested in the stool.

As such, the authors should assess whether Salmonella found in the stool express curli protein. Moreover, the authors should test whether limiting the ingestion of shed stool (using metabolic cages) reduces or eliminates the presence of curli +ve Salmonella in the intestines of infected mice. Even if this is the case, the study is still interesting, but it will clarify if the intestinal environment itself can induce curli expression.
RESPONSE: This is an important point. We show in the manuscript that the curli is expressed in the feces. We agree with the reviewer and included in the Discussion section that the expression of curli in the feces may be contributing to the levels of Salmonella transmission and thereby may consist of an important step in the pathogenesis of disease. However, we believe at this point a full implementation of transmission studies are beyond the scope of this paper. We would like to study this phenomenon further in a future study.

We do not expect that elimination of stool ingestion would decrease the curli expression. During our experiments we observed that even if we inoculate the animals with S. Typhimurium that expressed curli as detected by bioluminescence, no bioluminescence was detected at day 1 and day 2. However, bioluminescence was detected at day 3 or 4 after infection suggesting that curli expression was turned down during the initial stage but later turned on at later stages of infection. We suspect that there may be signals in the intestinal tract that regulates curli expression during inflammation. Consistent with this idea, Dr. Gunn’s group at OSU showed that bile may be one of these signals. However, the effect of secondary bile metabolites generated during S. Typhimurium infection or their combination with other metabolites on curli expression needs to be carefully studied. We plan to conduct these studies in the future.

2. Figures 5 and 6 - evaluation of inflammation in the joints. Considering that the majority of the readers of PLoS Pathogens will know little if anything re: joint inflammation – the data presented seems less than impressive (ie. a pathology score and some histology). I would request that the authors provide more detailed analysis – ie. immunostaning for inflammatory cells, proliferation etc, so that readers will get a better understanding of the processes involved.

RESPONSE: We agree and thank the reviewer for this comment. We have consulted with our colleagues who are experts in Rheumatology, (Drs Phil Cohen, Marc Monestier and Roberto Caricchio). All of them advised us that the most accurate way to evaluate joint inflammation and arthritis is histopathology. We suspect that either the direct presence or antigen curli or indirect damage by autoantibodies or a specific type of immune response (Th17 or type I interferon mediated) could be causing the inflammation in joints. As the possibilities are many, we hope the reviewer appreciates that these studies will take too long to address.

Reviewer #2: The inclusion of a curli mutant and a complemented strain is important in the experiment described in Figure 1.

RESPONSE: See our response to Reviewer’s comment above.

2-Anti-curli IgAs should be assayed (in addition to IgGs) in intestinal contents of infected mice.

RESPONSE: See our response to Reviewer’s comment above.

3-Titers of curli and ds-DNA Abs should be determined, and not "Arbitrary Units".

RESPONSE: See our response to Reviewer’s comment above.

4- Experiments are needed to clarify if mice in which light was not detected (lack of csgD expression) (Fig. 2) produced or not Abs against dsDNA.

RESPONSE: CsgD is expressed in a bistable manner, meaning that there are cells that are CsgD-ON and cells that are CsgD-OFF and very few cells with expression in between (Grantcharova et al., 2010, PMID: 19897646; MacKenzie et al., 2015, PMID: 25824832). The lack of detectable expression in some individual mice could simply mean that there are not enough cells in one concentrated area to generate detectable light production, or it could represent a truly negative population based on specific host-pathogen interactions, in which case we would expect that these mice would produce fewer dsDNA-antibodies. We agree with the reviewer that this would be interesting to investigate. However, in this study, the statistical analysis accounts for variability between different mice and showed a significant difference between mice infected with curli-positive or curli-negative Salmonella (Fig. 4E, 5C).

The protocol for isolation of curli from bacteria is rather complex and lengthy. However, the authors detect curli in different regions of the gut with formic acid alone. Negative controls including tissues from a mouse infected with a curli mutant and from a naive mouse should be included. Since the Enterobacteriaciae produce curli it is important to discriminate between mouse microbiota curli and the Salmonella curli.
RESPONSE: The purification procedure for curli essentially involves digesting or extracting away all of the soluble components of the bacterial cells, leaving behind insoluble proteins and polysaccharides. This is where the curli fibers are purified from (Collinson et al., 1991, PMID: 1677357). It is the same for the whole tissue samples from mice that were colonized by Salmonella. The first step in the procedure was to boil the ground up tissue samples in SDS-PAGE sample buffer, extracting the majority of soluble proteins from the sample and leaving the insoluble fraction behind. This is where the curli fibers were detected from. We have added sentences towards the end of the section “Curli are produced by *S.* Typhimurium after oral infection of antibiotic-treated or untreated mice” to explain this aspect better. The insoluble fraction was resuspended in 90% formic acid, frozen and lyophilized and this step was repeated 3 times in total. We standardized the tissue samples by weight (i.e., if the sample was 60 mg, it was resuspended and boiled in 600 uL of sample buffer and the insoluble portion was resuspended in 600 uL of formic acid x 3 treatments; if the sample was 55 mg, then the volumes were 550 uL). It should be noted that once curli fibers are depolymerized by formic acid treatment, the samples are somewhat fragile, since individual curli subunits are prone to re-aggregation (see Collinson et al., 1991). This makes these samples specialized.

In Figure 1, the curli staining is co-localized with Salmonella-specific staining, giving strong evidence that the Salmonella are the predominant bacterial species that are producing curli. However, we agree with the reviewer and it also occurred to us that another bacterial species/strain present in the normal mouse microbiota could be producing curli. To test for this possibility, we screened colon samples from 8 control mice that do not contain Salmonella. These blots are shown below and have been added to the manuscript as Supplementary Figure 4. The images at the top represent blots with automatic exposure of the Odyssey CLx imaging system, showing clearly the monomer and dimer CsgA species in the pure curli control and nothing in the colon samples. The images at the bottom represent the same blots over-exposed to show that no faint curli bands were evident in any of the control colon samples.

Reviewer #3: 7. Figure 3 C western blot is missing a loading control. This panel should probably be replaced so that the data can be properly quantitated.

RESPONSE: Due to the uniqueness of the curli-containing samples, as described above, they are not amenable to standard quantitation or normalization procedures. All of the proteins used as loading controls (actin, gapdh, tubulin, H3 etc.) are highly soluble, so would have been removed in the initial lysis before the insoluble pellet was treated with formic acid. As a result they can’t be used as loading controls. It’s possible that a protein could be spiked into the formic acid, but in our opinion this wouldn’t improve standardization.

8. Figure 2 shows that csgD expression is detected in non-streptomycin treated animals. However, line 166-167 mentions that curli-specific bands were seen in only 2 out of 6 non-streptomycin treated mice.

RESPONSE: We have reported a correlation between higher CFU levels in feces (and by extension, higher levels in the lower GI tract) in strep-treated mice and a higher proportion of mice with curli detected. We don’t know if the lack of curli detection in untreated mice is due to 1) production of curli below detectable limits, or 2) tissue samples where Salmonella is not uniformly distributed, or 3) no curli being produced by Salmonella in these mice. To mitigate against 2), we screened at least two independent 50 mg samples from each tissue, representing ~20-40% of the total weight, and in 4 of 6 untreated mice, both samples were negative. It is difficult to resolve 1), since we estimate that Salmonella proteins represent less than 1% of the total protein content in the ground up tissue samples. Likely the only reason we were able to detect curli is because it is an amyloid and is incredibly stable. We won’t be able to fully resolve the differences between strep-treated and untreated mice until we are able to track the fate of individual cells in vivo. We will attempt to do this in the future using Salmonella reporter strains.

9. Figure 4C immunoblots are not very clear and it is somewhat difficult to parse out the main points.

 RESPONSE: We apologize for this. Now we adjusted the light and contrast in these images.

Part III – Minor Issues: Editorial and Data Presentation Modifications
Please use this section for editorial suggestions as well as relatively minor modifications of existing data that would enhance clarity.


Reviewer #1: The authors indicate that the lack of luciferase expression under the csgD promotor by Salmonella in the liver and spleen indicates that systemic Salmonella do not express the gene. This is incorrect. Luciferase detection depends on many factors, including whether sufficiently large numbers of Lux+ve bacteria are nearby each other. Thus, Salmonella may be expressing the gene csgD at systemic sites, but based on their relatively low density, they cannot be detected. As such, the authors comments should be revised.
RESPONSE: We agree with the reviewer about the limitations of detecting luciferase activity. We revised the text and added this information. It now reads “It is important to note that sufficiently large numbers of luciferase positive bacteria are needed to detect the light production. Therefore, S. Typhimurium may also be expressing the csgD gene at systemic organs, but at relatively low density that cannot be detected with this technique.”

We did not detect curli associated with any of the Salmonella cells we observed in the liver; Figure 1E and H are representative images.

Line 499 – spelled jejunum incorrectly ie. “Two halves of the small intestine (i.e., jejenum and ileum)”
RESPONSE: Corrected.

Reviewer #2: Fig. 4C. Western blots should be improved. They are too dark and difficult to assess what is positive or negative.
RESPONSE: Corrected.

Reviewer #3: 1. Line no. 35-38. The first half of the sentence is corroborated by the experiments done in this manuscript while the second half has not been addressed.

RESPONSE: Yes this is the implication of our study. We kept this in the abstract due to the recent publications of curli’s importance on gut-brain axis.

2. Supplementary Fig. S2B, scale bar is missing.

RESPONSE: Corrected.
3. Figure 1E, H- The supplementary figure S1A shows colonization of the liver following oral infection of S. Typhimurium cells. However almost no bacterial cells are seen in the immunohistochemistry staining. Given the techniques, is this expected or are the particular pictures misleading?

RESPONSE: This is expected. Although S. Typhimurium is present, it is difficult to detect the bacteria in each of the sections from the liver using immuno-histochemistry. Detecting bacterial numbers using plating is a more accurate way to detect bacterial loads. We can confirm that the images are representative, as we scanned as much of the liver as we could.

4. Figure 2 A, B. There are four panels for each of the condition tested. Do these panels represent replicates? Please clarify in the legend.

RESPONSE: We have changed this in the figure legend. Each panel represents a different mouse.

5. Line 144-146 (Figure 2 A, B) Some of the GI tracts of the infected mice do not show any light production. In addition, there seems to be lot of variation in the expression of csgD in the GI tract of the infected animals. Could the authors comment on the variation?

RESPONSE: Please see our response above.

6. Figure 3C. The authors collect tissue sample from infected mice and probe them for the presence of curli amyloid fibers. To depolymerize the fibers, the authors treat the tissue sample three time with 90% formic acid (FA). These three successive FA treatments are somewhat unique, as I have not seen repeated FA treatments used in other protocols. Since FA is known to fragment proteins the laddering effect seen in figure 3C western blot might be caused by the repeated FA treatments. The authors might try another denaturant.
RESPONSE: We agree with the reviewer, it would be nice to have tested another method to depolymerize curli fibers. However, the only widely used treatment that has been published is treatment with 90% formic acid.

10. Figure 4D, the data from ELISA for CsgA specific IgG production could be represented in the same format as figure 4E which shows the levels of anti-dsDNA autoantibodies in WT vs csgBA mutant infection. This would make it easier for the reader to compare the effect of presence vs absence of curli on host immune response.

RESPONSE: The reason that the ELISA for CsgA specific IgG production was presented in its current format is to show that these antibodies are specific and can be diluted. This is a standard method used to show specificity as the protocol was developed in the lab and the specific antibody levels are low.

11. Figure 5B- Its not clear what the two images represent. Could the relevant description be added to figure legend?

RESPONSE:Corrected

12. Line 239-240. The data for the statement is not present, but should be added.

RESPONSE:Corrected

13. Figure 6F, it’s not clear what the two “E. coli” data points represent in the figure.

RESPONSE: Figure is revised now.
